# Growing *Staphylococcus aureus* in synthetic cystic fibrosis medium promotes colonization in a murine pneumonia model

**DOI:** 10.1128/iai.00283-26

**Published:** 2026-08-06

**Authors:** Justin M. Luu, Ian N. Moore, Dina A. Moustafa, Joanna B. Goldberg

**Affiliations:** 1 Microbiology and Molecular Genetics Program, Graduate Division of Biological and Biomedical Sciences, Laney Graduate School, Emory University 1371, Atlanta, Georgia, USA; 2 Department of Pediatrics, Division of Pulmonary, Asthma, Cystic Fibrosis, and Sleep, Emory University School of Medicine 12239, Atlanta, Georgia, USA; 3 Department of Pathology and Laboratory Medicine, Emory National Biomedical Research Center, Emory University School of Medicine 12239, Atlanta, Georgia, USA; 4 Emory+Children’s Center for Cystic Fibrosis and Airway Disease Research, Emory University School of Medicine 12239, Atlanta, Georgia, USA; University of California Davis, Davis, California, USA

**Keywords:** *Staphylococcus aureus*, mouse model, cystic fibrosis

## Abstract

*Staphylococcus aureus* is a leading cause of bacterial infections worldwide and can lead to diseases, such as osteomyelitis, skin, and lung infections in humans. Murine infection models have been an essential tool to the study of *S. aureus* virulence, although they do have their limitations. In murine pneumonia models, *S. aureus* infections are generally not well maintained. We hypothesize that culturing *S. aureus* under different conditions, such as in Synthetic CF Medium 2 (SCFM2), would increase bacterial respiratory loads compared to growing the bacteria on standard rich medium agar plates. We observed that culturing six *S. aureus* strains from various sources in SCFM2 resulted in a neutral or increased level of bacterial lung load compared with those cultured on agar plates. Focusing on one strain, WU1, we found that culturing in SCFM2 improved bacterial persistence in the oropharynx and led to a sustained long-term lung infection compared with agar plates. We further noted that mice experienced increased inflammation in both lobes of the lungs when infected with WU1 cultured in SCFM2 compared with agar plates. Overall, we have shown that culturing *S. aureus* under different conditions prior to infection impacts the level of colonization and host response.

## INTRODUCTION

*Staphylococcus aureus* is a Gram-positive opportunistic pathogenic bacterium and a leading cause of bacterial infections worldwide (1). This bacterium can colonize a variety of mammals, including humans (2). In humans, *S. aureus* can persist in multiple anatomical sites and can cause a wide variety of infections, such as osteomyelitis, endocarditis, skin, and lung infections (3). Additionally, approximately 30% of the human population is asymptomatically colonized with *S. aureus* in their nasal cavity (4).

Murine models, particularly mice, have been an essential tool to study *S. aureus* in the context of infections (5). They offer many advantages, such as small size, relative ease of breeding, cost efficiency, a well-characterized immune system, and the ability to generate transgenic strains. For these reasons, mice have been used to study infections induced by *S. aureus,* such as sepsis, osteomyelitis, endocarditis, and pneumonia (6–9). However, in murine pneumonia models, there are difficulties with maintaining bacterial colonization, which complicates assessing potential treatments (10). In mouse models of *S. aureus* pneumonia, most studies have had to administer supraphysiological (10^7^–10^8^ colony-forming units (CFUs)) doses intranasally to establish infection (9–13); doses below this threshold generally result in clearance rather than a sustained infection.

To improve the sustainability of *S. aureus* during murine pneumonia infections, modifications to the bacterial preparation and the mouse strains used have been explored. Intratracheal administration of *S. aureus* embedded in agar beads to mice has led to prolonged infections for at least 21 days (14, 15). Cyclophosphamide, an immunosuppressive chemotherapy that leads to the depletion of neutrophils, has also been used to render mice immunocompromised and more susceptible to *S. aureus* infections (16–18). Additionally, the choice of bacterial strain used in these studies has been evaluated. Mouse-derived *S. aureus* strains have been shown to more persistently colonize the murine nasopharynx compared with human-derived *S. aureus* strains (19, 20). These approaches have been greatly beneficial to advancing *S. aureus* infection models; however, these modifications have important limitations, which have restricted their adoption. In particular, while embedding *S. aureus* in agar beads improves infection sustainability, it does not accurately represent a clinical infection for diseases such as cystic fibrosis (CF).

CF is a multi-organ genetic disease that impacts more than 120,000 people worldwide (21). This disease is caused by mutations in the CF transmembrane regulator (CFTR), a sodium and bicarbonate ion channel, which negatively impacts mucus clearing in the lungs and leads to the buildup of sputum (22). Microbial pathogens can utilize sputum as a nutrient source to establish and maintain chronic infections (23). As a result, CF patients often experience persistent, lifelong infection with respiratory pathogens in their lungs (24). These chronic bacterial respiratory infections remain the leading cause of morbidity in these patients even with the advent of CFTR modulator therapies. While these therapies have improved the quality of life of these patients, they do not completely eradicate infections caused by bacterial respiratory pathogens, such as *S. aureus* (25). *S. aureus* is the most frequently isolated bacteria from respiratory samples of CF patients (8) and persistently colonizes the lungs of this population (26, 27). Thus, models to study *S. aureus* in the context of lung infections are paramount to understanding its impact in CF infections.

Here, we studied the growth of *S. aureus* under two different culture conditions prior to infection and monitored its impact on bacterial respiratory load and host response. Traditionally, to prepare *S. aureus* for infection, the bacteria are cultured in rich laboratory media (such as tryptic soy or lysogeny) on plates or in broth prior to infection (28–30). Rich media is relatively inexpensive, widely used and studied, and offer consistency between laboratories. However, this growth condition does not replicate the nutrient composition of a human environment. To address this issue, a defined laboratory medium that mimics the nutrient composition of sputum from CF patients was developed called Synthetic CF Medium 2 (SCFM2) (31, 32). A previous study by Ibberson and Whiteley revealed that when comparing the gene expression of *S. aureus* cultured in rich media to human CF sputum, the rich medium transcriptomes clustered independently of human samples (33). Furthermore, they observed that when cultured in SCFM2, the *S. aureus* transcriptomes were more similar to *S. aureus* from human CF sputum compared with rich laboratory media (33). This similarity was only achieved when SCFM2 was supplemented with a stimulus from neutrophils to activate the SaeR/S two-component system, a system that is also activated by mouse neutrophils (34). We hypothesize that the mouse lung environment would mimic the neutrophil-mediated effect needed to activate the SaeR/S system. Here, we propose that the choice of environmental conditions to grow *S. aureus* prior to infection may improve bacterial respiratory load during murine pneumonia models and hypothesize that culturing *S. aureus* in SCFM2 prior to infection will increase the bacterial load and persistence in the lung.

To test this hypothesis, we cultured six different *S. aureus* strains on rich media (lysogeny broth; LB) agar plates and in SCFM2. These strains were then administered to immunocompetent mice, and the bacterial respiratory loads were determined. We observed that while the effects are strain-specific, culturing in SCFM2 resulted in a neutral or increased level of the bacterial burden or load in the lung. With WU1, a *S. aureus* strain known to persistently colonize the oropharynx in mice, we discovered that culturing in SCFM2 increased bacterial persistence in the oropharynx and led to a sustained level of bacterial lung load for at least 4 days. Furthermore, infections with this strain cultured in SCFM2 increased inflammation and altered cytokine levels in both the left and right lung lobes compared with infections with this strain grown on agar plates. Overall, we demonstrate the impact of culturing *S. aureus* in SCFM2 compared with agar plates on both bacterial load and host response in a murine pneumonia model.

## RESULTS

### *S. aureus* strains used in this study exhibit both clinical and genomic diversity

**Fig 1:**
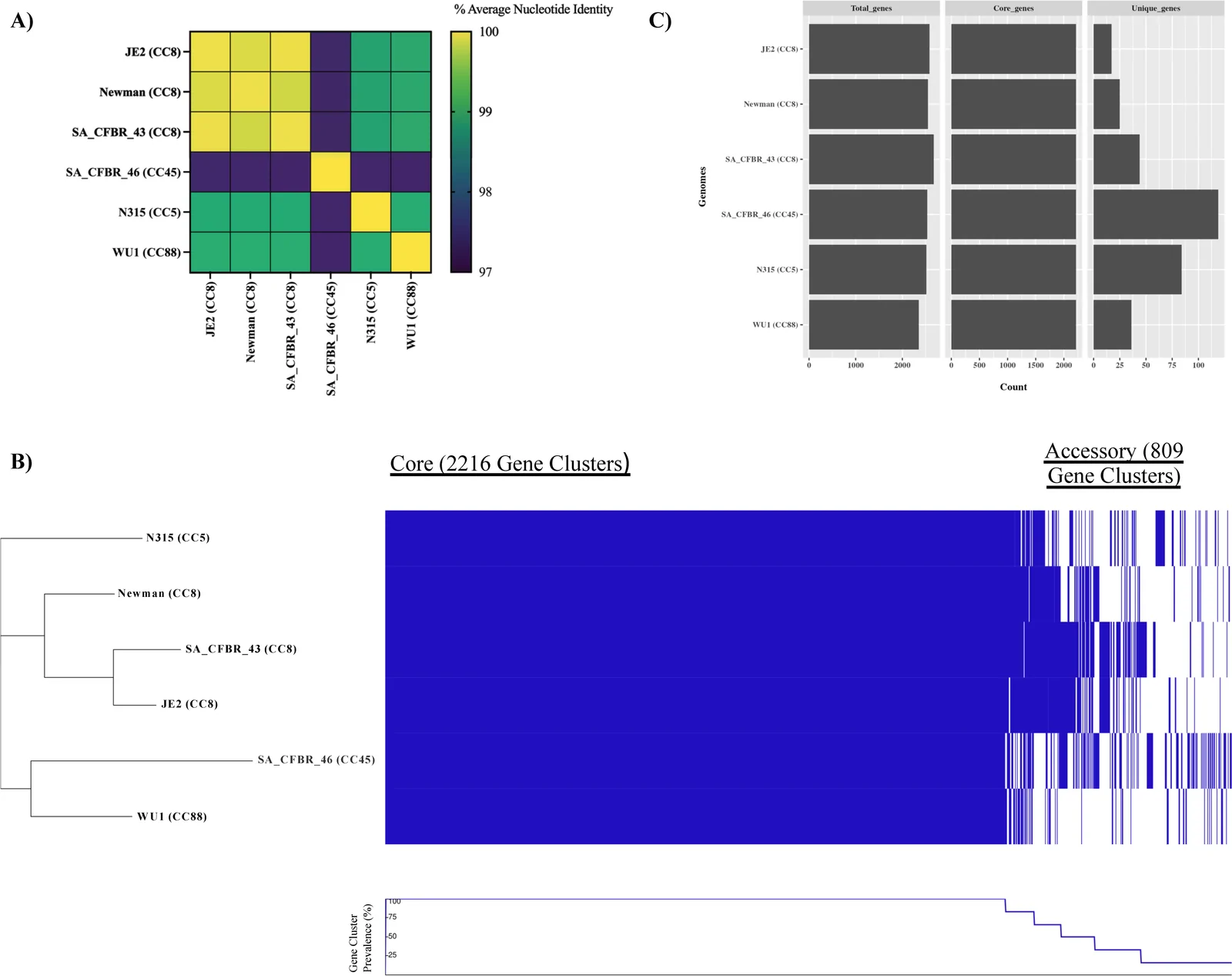
Strains used in this study exhibit genetic diversity. The genomic sequences of each *S. aureus* were compared with each other. (**A**) Average nucleotide identity between the strains were determined with pyani. The numeric results (Table S1) were used to generate the heatmap. (**B**) Phylogenetic analysis of the strains was performed with PIRATE. The phylogenetic tree, core genome, and accessory genome were visualized using Phandango. (**C**) The number of unique genes for each strain was extracted from the phylogenetic analysis and plotted with ggtree.

The goal of this study is to characterize whether altering culture conditions impacts *S. aureus* bacterial load levels during murine pneumonia. To achieve this, we sought to test a diverse set of *S. aureus* strains under two growth conditions. We used six *S. aureus* strains: five strains isolated from human infections and one strain isolated from a mouse infection (Table 1). In addition, the strains came from varying clonal complexes (CCs), a grouping system based on the similarity of specific housekeeping genes. In the United States, CC8 and CC5 are the dominant strains isolated from patients, and we included strains to represent both (35). JE2 and Newman, both CC8 strains, are frequently used as model strains for methicillin-resistant (MRSA) and methicillin-sensitive (MSSA) strains, respectively (36, 37). JE2 is a derivative of a strain isolated from a skin abscess, while Newman was isolated from a tubercular osteomyelitis infection. N315, an MRSA pharyngeal isolate, was chosen as a representative CC5 strain (38). Sa_CFBR_43 (MRSA) and Sa_CFBR_46 (MSSA) were isolated from respiratory samples of two different patients with CF (39). Sa_CFBR_43 belongs to CC8, while Sa_CFBR_46 belongs to CC45, a lineage of *S. aureus* that has two classes of *S. aureus* quorum sensor regulators (40). WU1 (MSSA) was isolated from an outbreak of preputial gland infections in a mouse breeding facility and belongs to CC88, a CC that is primarily isolated from rodents in North America and Europe (20, 41). We determined the diversity of these strains genomically through average nucleotide identity analysis (Table S1) (Fig. 1A). Perhaps not surprisingly, JE2, Newman, and Sa_CFBR_43, all being CC8, shared greater than 99.9% similarity. Sa_CFBR_46 was the most genetically distant compared with the other strains, followed by WU1. Phylogenetic analysis revealed that the strains share a core genome of 2,216 genes and an accessory genome of 809 genes (Fig. 1B). Sa_CFBR_46 contained the largest number of unique genes (119 genes), followed by N315 (84 genes), Sa_CFBR_43 (44 genes), WU1 (36 genes), Newman (25 genes), and finally JE2 (17 genes) (Fig. 1C).

**TABLE 1:** *Staphylococcus aureus* strains utilized in this study

Strain	Mrsa/ MSSA	Clonal complex	Origin	Host	Reference
JE2	MRSA	8	Skin abscess	Human	(36)
Newman	MSSA	8	Tubercular osteomyelitis	Human	(37)
Sa_CFBR_43	MRSA	8	Cystic fibrosis respiratory sample	Human	(39)
Sa_CFBR_46	MSSA	45	Cystic fibrosis respiratory sample	Human	(39)
N315	MRSA	5	Pharyngeal	Human	(38)
WU1	MSSA	88	Preputial gland abscess	Mouse	(20)

### *S. aureus* strains exhibit comparative growth kinetics in SCFM2 and LB

**Fig 2:**
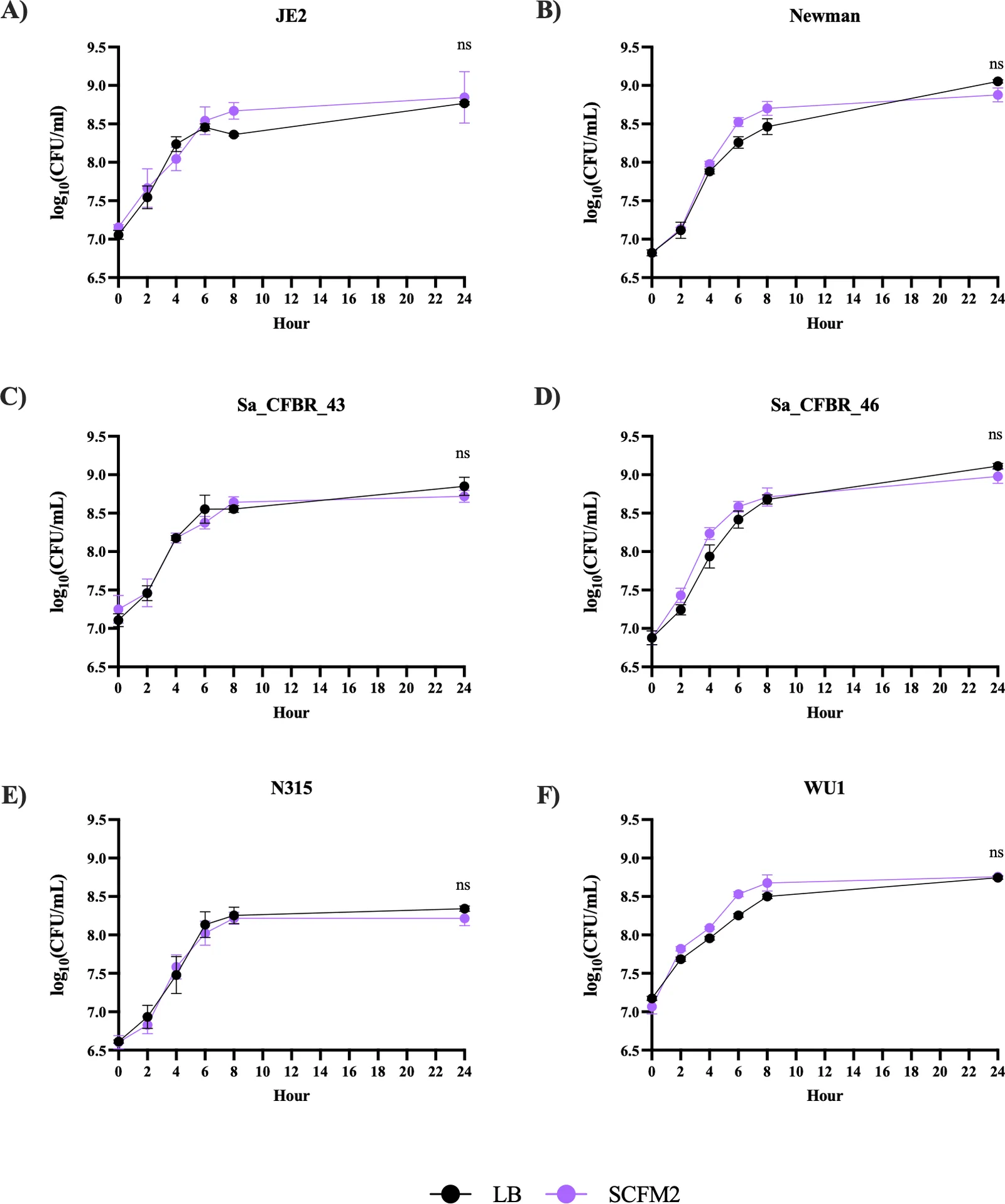
*S. aureus* strains exhibit comparative growth kinetics in SCFM2 and LB. JE2 (**A**), Newman (**B**), Sa_CFBR_43 (**C**), Sa_CFBR_46 (**D**), N315 (**E**), and WU1 (**F**) were grown statically in either LB or SCFM2 in a 6-well plate at a starting OD_600_ of 0.01. Aliquots were removed, serially diluted, and plated on LA plates at 0, 2, 4, 6, 8, and 24 h. The results are plotted as the mean of biological triplicates with standard deviation. Statistical analysis was performed on the 24-h time point through unpaired Student’s *t*-test with Welch correction. ns = not significant.

LB and SCFM2 differ in nutrient composition, which could have an impact on *S. aureus* growth and physiology (31). To determine whether this difference impacts growth, all strains were cultured statically in either LB or SCFM2 in six-well plates and sampled for CFUs at 2, 4, 6, 8, and 24 h (Fig. 2). During the first 8 h of growth, all strains exhibited similar growth kinetics in both media. At 24 h, there was an equal number of CFUs for all strains cultured in both media. Under the experimental conditions of this study, our results indicate that the choice of culture media, LB or SCFM2, does not substantially alter the growth kinetics of the strains investigated here.

### Culturing *S. aureus* in SCFM2 increases the bacterial load in the lung for most examined strains

**Fig 3:**
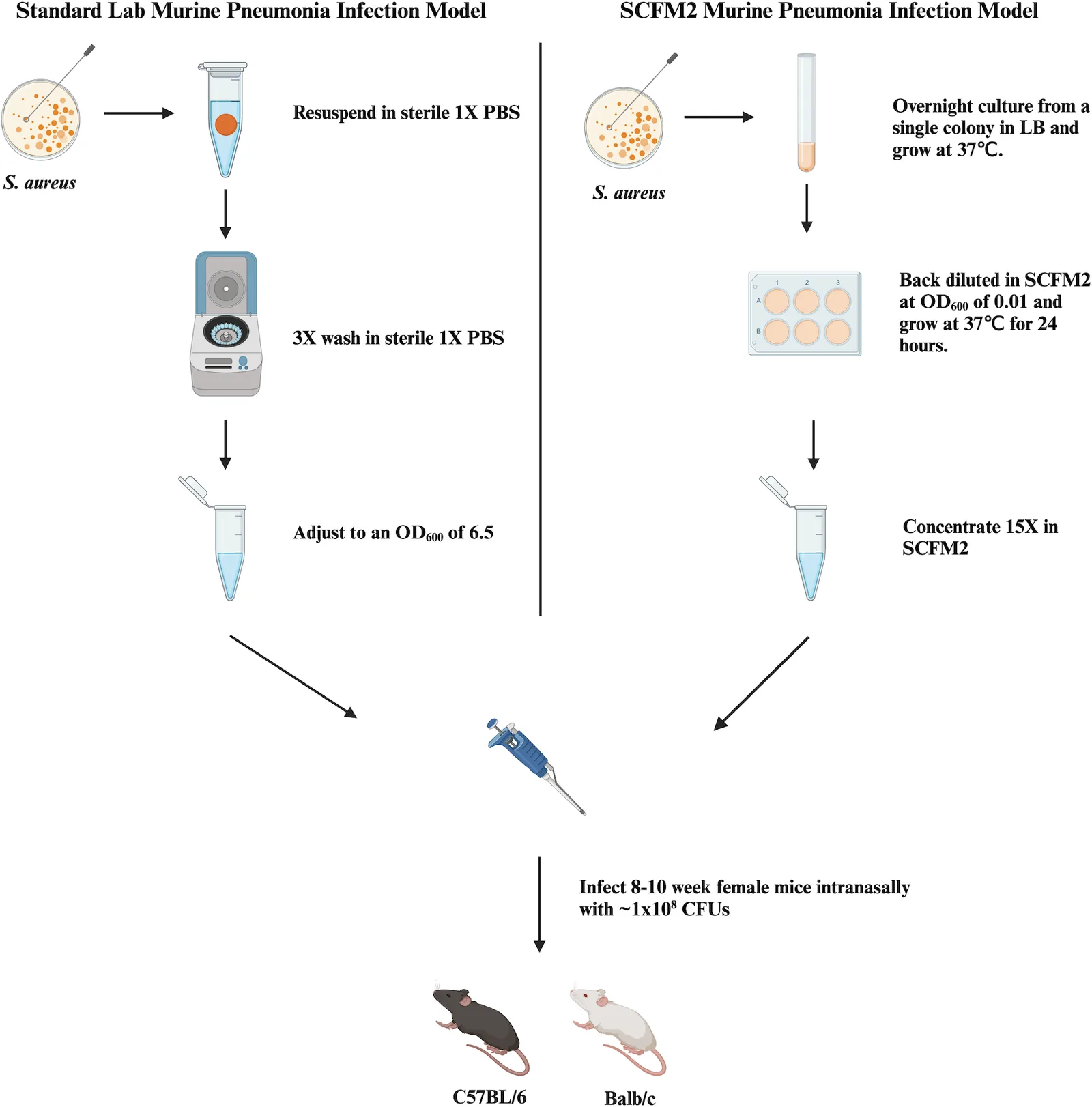
Schematic of the *S. aureus* preparation workflows used in this study. *S. aureus* strains were cultured either on agar plate (left) or in SCFM2 (right). For the agar plate preparation, *S. aureus* was grown on *Staphylococcus* Isolation Agar (SIA) overnight at 37℃, swabs of colonies were transferred to 1× PBS, washed three times in 1× PBS, and adjusted to an OD_600_ of 6.5. For the SCFM2 preparation, a single colony from *S. aureus* grown on SIA was transferred to LB and grown overnight shaking at 37℃. The culture was washed three times in 1× PBS, back diluted in SCFM2 at a starting OD_600_ of 0.01 in a six-well plate, grown statically for 24 h, and concentrated 15× in fresh SCFM2. Then, 12.5 µL of each condition was subsequently administered intranasally into each nostril (25 µL total corresponding to ~1 × 10^8^ CFUs) of anesthetized C57BL/6 or Balb/c mice.

**Fig 4:**
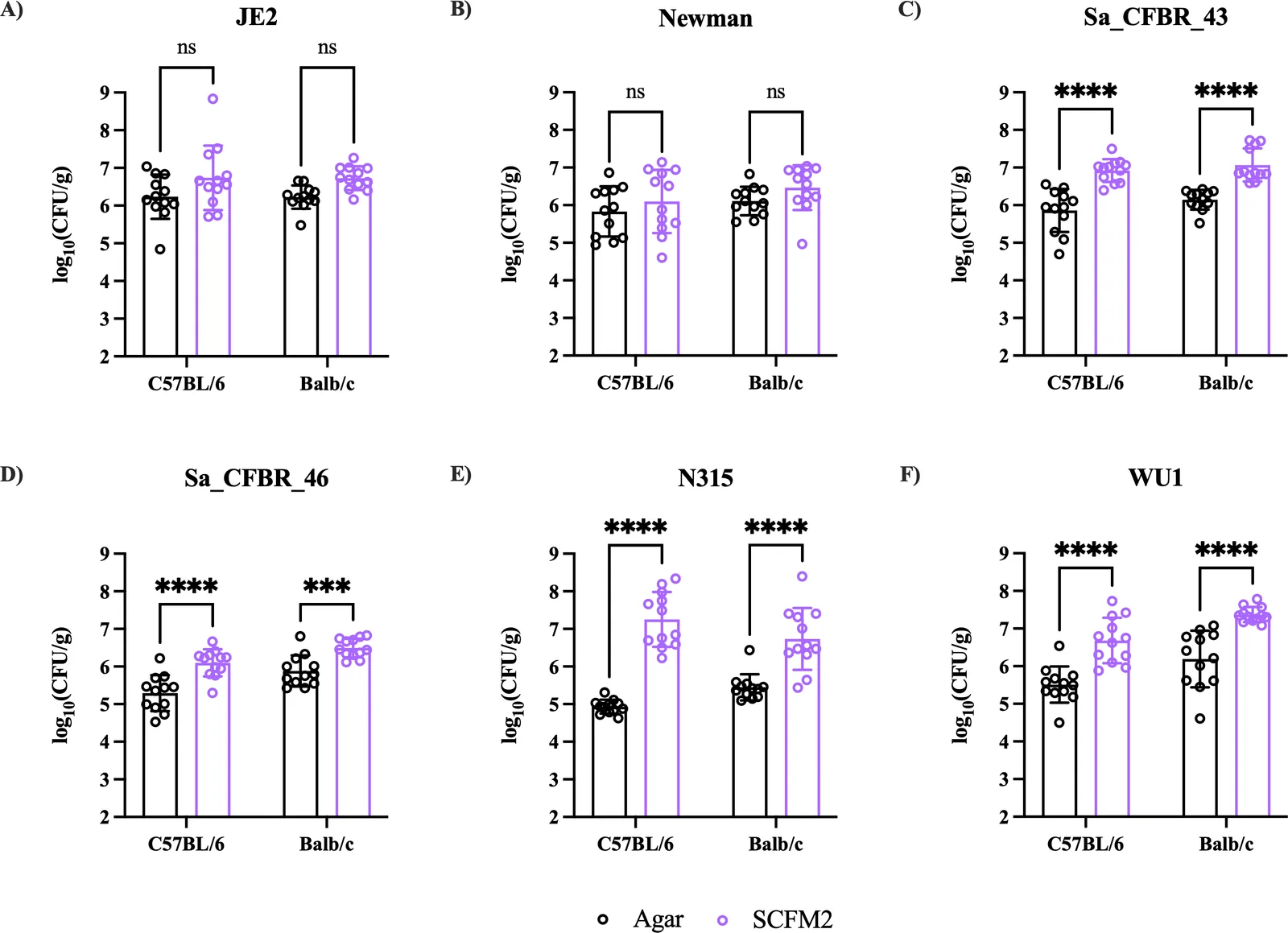
Culturing *S. aureus* in SCFM2 generally increases lung bacterial loads in mice during acute pneumonia independent of mouse strain. JE2 (**A**), Newman (**B**), Sa_CFBR_43 (**C**), Sa_CFBR_46 (**D**), N315 (**E**), and WU1 (**F**) were cultured on agar plates or in SCFM2 as described in Fig. 3 and administered to either 8–10-week-old female C57BL/6 or Balb/c mice. Mice were euthanized 24 h post-infection, the lungs were aseptically removed and weighed, homogenized in 1× PBS, and plated on SIA. Each data point represents a mouse, and each condition had a total of 12 mice. The *y*-axis was set to 10^2^, the limit of detection. The mean and standard deviation are represented by the error bars. Data were analyzed using two-way ANOVA with Šídák correction. ***P < 0.001, ****P < 0.0001, ns = not significant.

**Fig 5:**
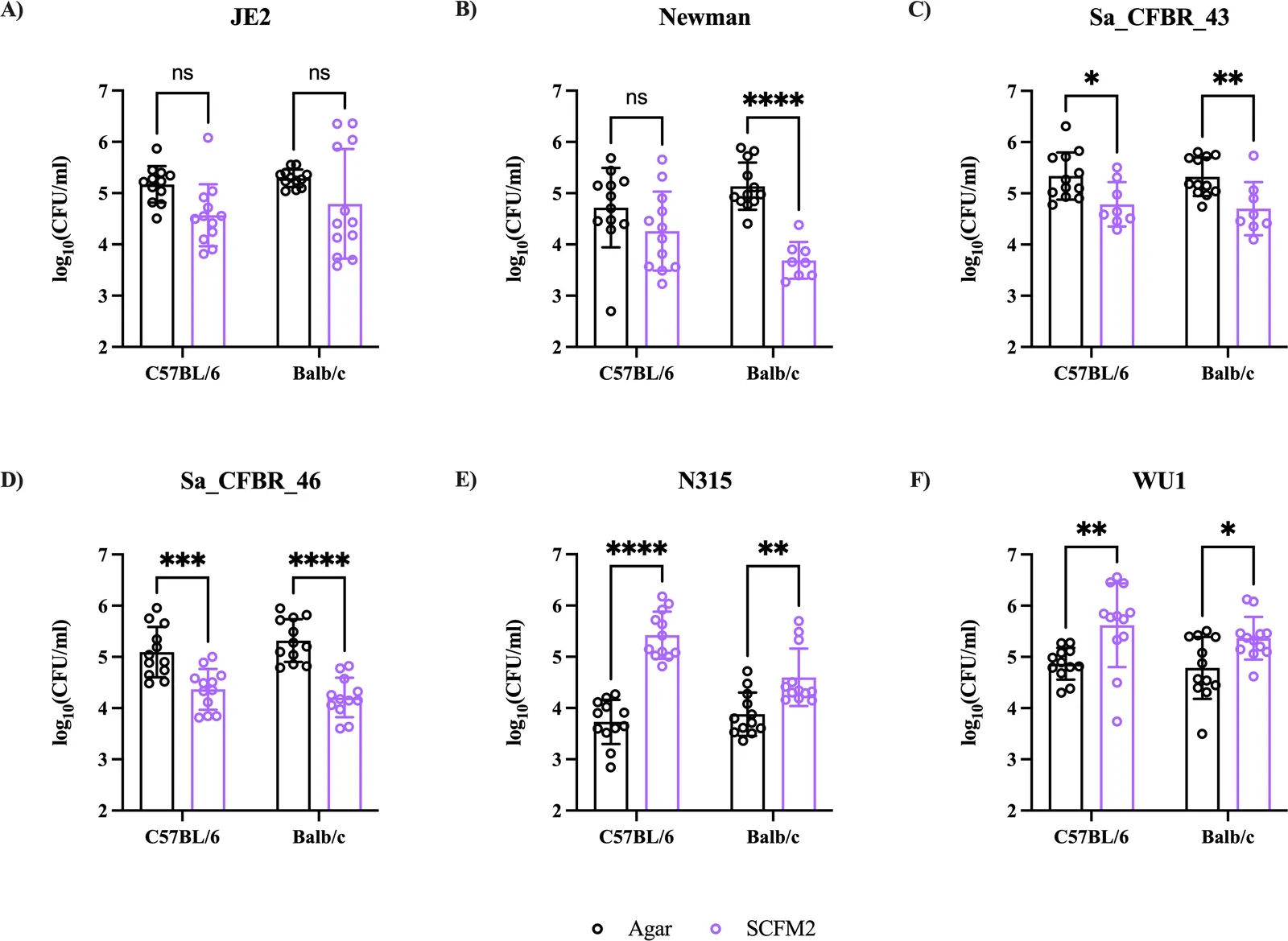
Impact of different culture conditions on nasal cavity bacterial load in mice during acute pneumonia. JE2 (**A**), Newman (**B**), Sa_CFBR_43 (**C**), Sa_CFBR_46 (**D**), N315 (**E**), and WU1 (**F**) were cultured either on agar plates or in SCFM2 as described in Fig. 3 and administered to either 8–10-week-old female C57BL/6 or Balb/c mice. Mice were euthanized 24 h post-infection and 1 mL of sterile 1× PBS was flushed through the nasal passage using an 18-G catheter placed at the nasopharyngeal opening. The washes were serially diluted and plated on SIA. Each data point represents a mouse, and each condition had 12 mice. The *y*-axis was set to 10^2^, the limit of detection. Data were analyzed using two-way ANOVA with Šídák correction. The mean and standard deviation are represented by the error bars. *P < 0.05, **P < 0.01, ***P < 0.001, ****P < 0.0001, ns = not significant.

We hypothesized that culturing *S. aureus* in SCFM2 would increase the bacterial load level in the murine lung compared with LB agar. To test this hypothesis, we infected 8–10-week-old C57BL/6 or Balb/c mice with ~1 × 10^8^ CFUs of each *S. aureus* strain, prepared through our agar plate or SCFM2 workflow (Fig. 3). At 24 h post-infection, the mice were euthanized, and bacterial loads in the lungs (Fig. 4) and nasal cavity (Fig. 5) were determined.

For all strains tested, we observed that mice infected with *S. aureus* cultured in SCFM2 exhibited more severe signs of disease compared with those infected with *S. aureus* cultured on agar plates. Our results revealed that culturing Sa_CFBR_43, Sa_CFBR_46, N315, and WU1 in SCFM2 significantly increased bacterial lung loads in both C57BL/6 and Balb/c mice compared with culturing on agar plates (Fig. 4C, D, E and F). In contrast, there was no significant difference in bacterial lung load for JE2 and Newman between the culture conditions in either mouse strain (Fig. 4A and B). We observed the highest bacterial lung loads for N315 in C57BL/6 mice and WU1 in Balb/c mice when cultured in SCFM2 compared with agar plates. The effects of SCFM2 on the bacterial nasal cavity load were more variable (Fig. 5). While JE2 bacterial nasal passage load did not differ significantly in either mouse strain, Newman exhibited significantly lower bacterial nasal passage load in Balb/c mice when cultured in SCFM2 compared with agar plates but not in C57BL/6 mice (Fig. 5A and B). Both CF isolates, Sa_CFBR_43 and Sa_CFBR_46, had significantly lower bacterial loads in the nasal cavity of either mouse strain when cultured in SCFM2 (Fig. 5C and D). Finally, bacterial nasal passage loads in both N315 and WU1 were significantly higher when cultured in SCFM2 independent of mouse strains (Fig. 5E and F). Overall, our results indicate that while the effects of growing *S. aureus* in SCFM2 on lung and nasal cavity bacterial loads are strain-specific, it can improve bacterial load in the lungs at 24 h.

### Culturing WU1 in SCFM2 increases bacterial persistence in the throats and lungs

**Fig 6:**
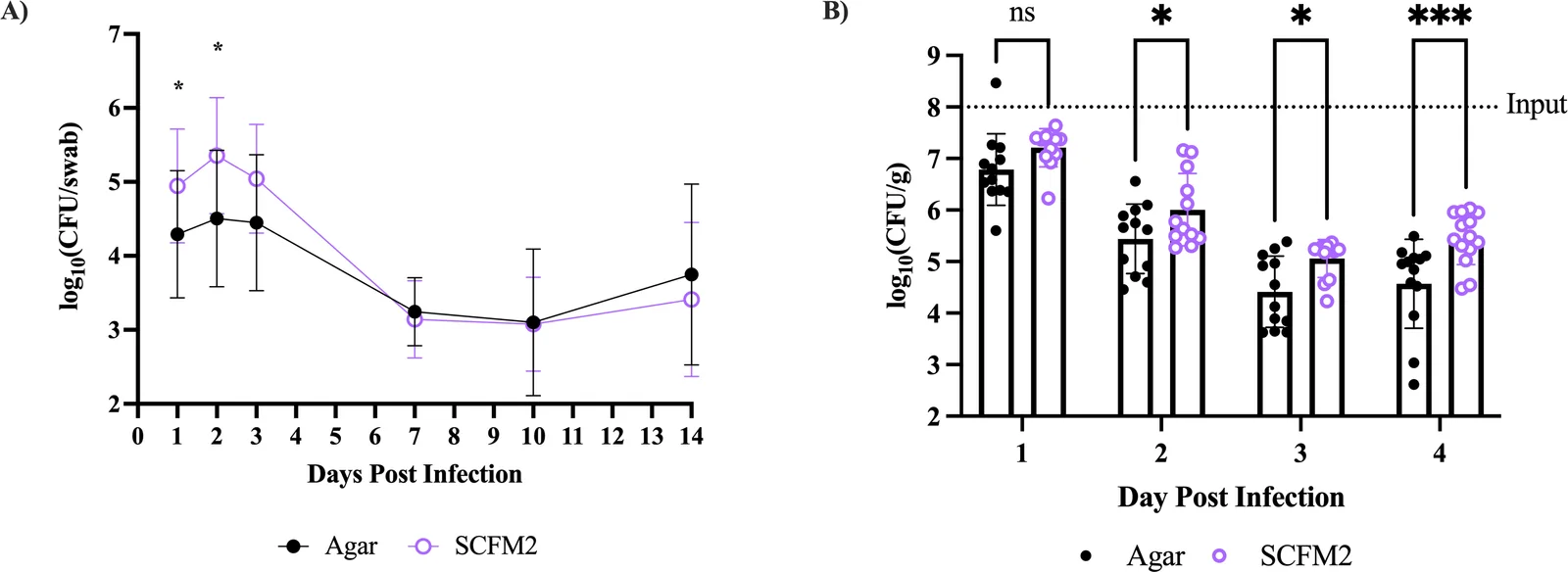
Culturing WU1 in SCFM2 increases bacterial persistence in the throats and lungs. *S. aureus* strain WU1 was cultured on agar plates or in SCFM2 as described in Fig. 3 and were administered to 8–10-week-old female Balb/c mice. (**A**) The throats of the same mice were swabbed on 1, 2, 3, 7, 10, and 14 days post-infection, the swabs were resuspended in 1× PBS, serially diluted, and plated on SIA. Each point represents the average and standard deviation of 12 to 15 mice. (**B**) A separate cohort of mice were euthanized after 1, 2, 3, and 4 days post-infection, the lungs were aseptically removed and weighed, homogenized in 1× PBS, and plated on SIA. Each data point represents a mouse, and each condition had a total of 12 mice. The *y*-axis was set to 10^2^, the limit of detection. Statistical analysis for the throat swabs was performed on the 1- and 2-day time point through unpaired Student’s *t*-test with Welsch correction. Multi-day lung colonization was analyzed using two-way ANOVA with Fisher’s LSD post-hoc test. *P < 0.05, ***P < 0.001, ns = not significant.

Next, we studied whether SCFM2 also influences the bacterial loads in nasopharynx and lungs over a longer infection. For this experiment, we selected WU1 and Balb/c mice because this combination resulted in consistently high bacterial loads in the lung at 24 h. Previous studies have only characterized WU1’s ability to persistently colonize mice oropharynx during respiratory infections (20, 42, 43), and we aimed to determine if SCFM2 can improve the level and persistence in both the throat and lung. We intranasally infected Balb/c mice with ~1 × 10^8^ CFUs of WU1 grown using either culture condition and monitored bacterial loads in both the throat and the lungs of infected mice over time (Fig. 6). In one cohort of mice, bacterial persistence in the throat was determined by swabbing the oropharynx of the same mice on days 1, 2, 3, 7, 10, and 14 post-infection (Fig. 6A). There was significantly higher bacterial load in the oropharynx when WU1 was grown in SCFM2 compared with on agar plates at days 1 and 2 post-infection. No significant differences were observed on days 3, 7, 10, and 14 post-infection. This suggests that the SCFM2-driven increase in WU1 bacterial loads in the oropharynx persists for up to 2 days post-infection. Next, we monitored WU1 colonization in lungs over time using a separate cohort of Balb/c mice (Fig. 6B). Every day for up to 4 days, bacterial lung loads were determined. We observed consistent and significantly higher levels of lung bacterial loads up to 4 days post-infection when WU1 was grown in SCFM2 compared with agar plates. Overall, our results indicate that, in addition to increasing bacterial lung loads during early infections, culturing WU1 in SCFM2 increases oropharynx persistence 2 days post-infection and leads to sustained bacterial lung loads for 4 days post-infection.

### Infection with WU1 cultured in SCFM2 leads to increased inflammation and altered cytokine profile compared with agar conditions

We next sought to determine how culturing WU1 in SCFM2 vs agar plates impacts the host response in our pneumonia model using histology and cytokine analysis. Balb/c mice were intranasally infected with ~1 × 10^8^ CFUs of WU1 prepared through either culture methods; 1× PBS, and SCFM2 alone served as “vehicle controls.” For these studies, mice were euthanized 24-h post-infection, and the bacterial load in each lung lobe was determined (Fig. S1A). We observed a relatively even distribution of CFUs in the left and right lung lobes of mice infected with WU1 cultured in SCFM2 (Fig. S1B). For WU1 cultured on agar plates, we observed slightly higher CFUs recovered from the left compared with the right lung lobes. Overall, we observed higher bacterial loads in both lung lobes when mice were infected with WU1 cultured in SCFM2 compared with agar plates.

We aimed to determine whether the growth conditions of WU1 prior to infection impact the response in the lung. To do this, we infected mice with WU1 cultured either on agar plates or in SCFM2 and used two parallel sets of mice for histology and cytokine analysis. Since it is currently unknown if *S. aureus* respiratory infections lead to different pathology or cytokine responses in the right or left lobe, we separated the lobes and used one for histopathology and the other for cytokine analysis to determine lung lobe to lobe variability (Fig. S1A). In the first set, the left lung lobe was processed for histology, while the right lobe was processed for cytokine analysis. In the other set, the opposite lobe was processed for histology and cytokine analysis. Thus, for all these comparisons, the left and right lungs were from different mice.

**Fig 7:**
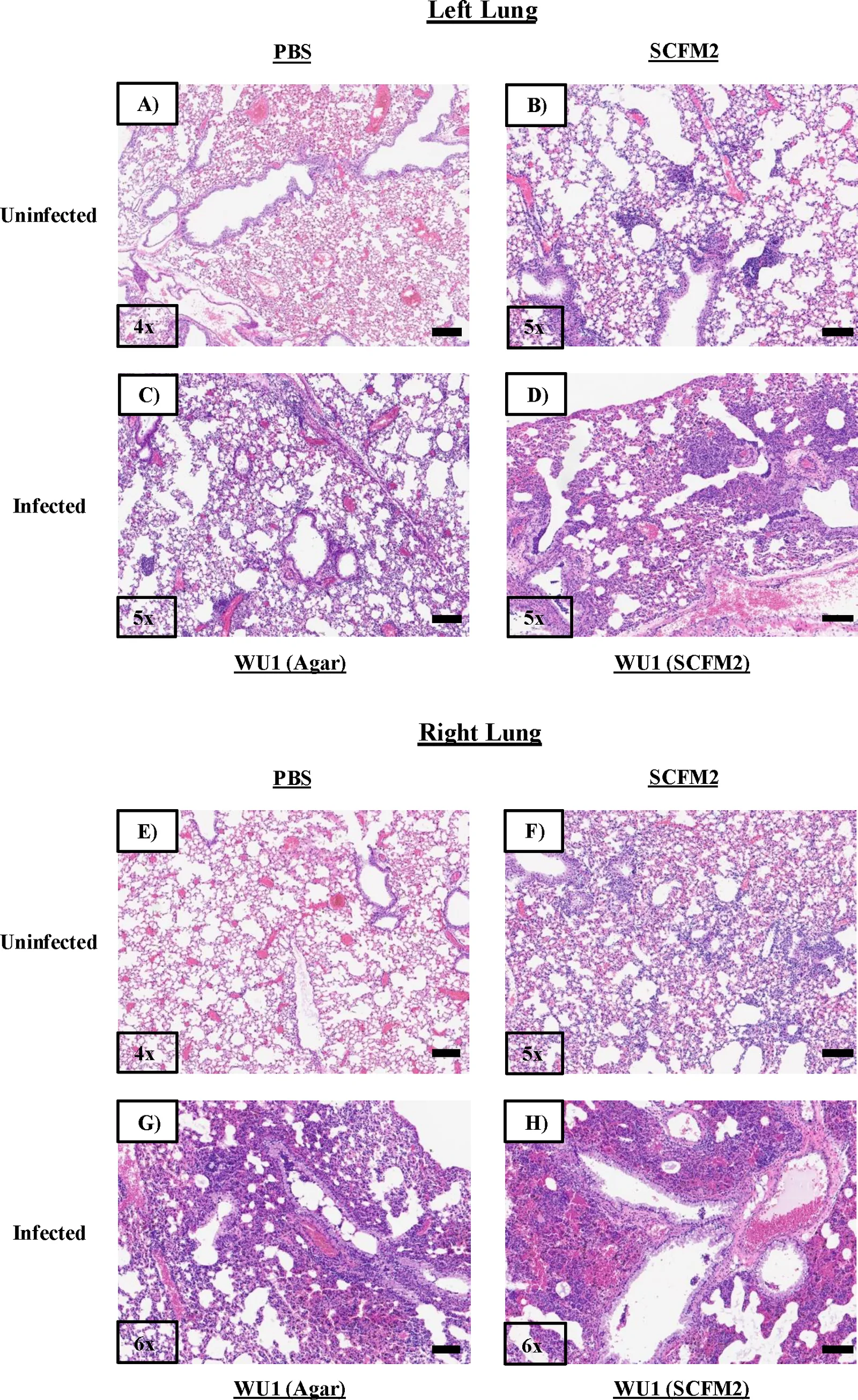
Infection with WU1 cultured in SCFM2 leads to increased inflammation and neutrophil recruitment in both lung lobes compared with agar plate preparation. *S. aureus* strain WU1, cultured either on agar plates or in SCFM2, as described in the workflow in Fig. 3, was administered to 8–10-week-old female Balb/c mice. Mice were euthanized 24 h post-infection. For each culture workflow (Fig. S1), the left lung (**A-D**) or right lung (**E-H**) lobes from each mouse (*n* = 6) were inflated and fixed using 4% neutral-buffered formalin (NBF). The lungs were then processed, stained with H&E, and blindly assessed by a board-certified veterinary pathologist. A representative image for each lung lobe is shown with the magnification stated on the bottom left (scale bars = 200 µm).

**Fig 8:**
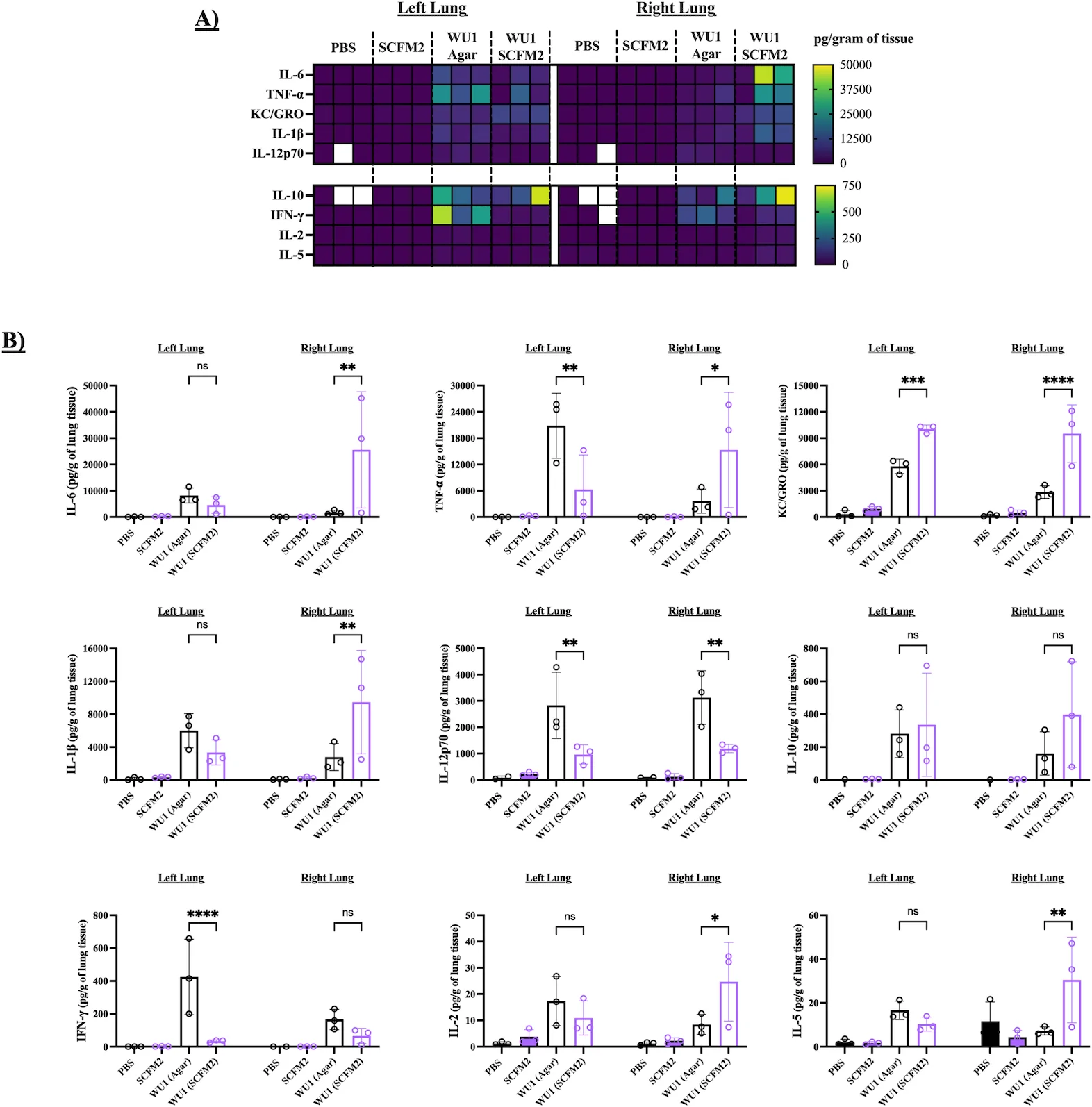
Cytokine profile from lungs infected with WU1 cultured on agar plates and SCFM2. *S. aureus* strain WU1, cultured either on agar plates or in SCFM2, as described in Fig. 3, was administered to 8–10-week-old female Balb/c mice. Mice were euthanized 24 h post-infection. For each culture workflow (Fig. S1), the left lung or right lung lobes were excised from each mouse (*n* = 6). The lungs were then homogenized in 1× PBS + 1× protease inhibitor, the cell-free lysates were isolated, and cytokines were measured with the V-plex proinflammatory cytokine panel 1 (MSD). Quantification of each cytokine was normalized to the weight of the lungs and visualized in a heatmap. Values below the limit of detection were represented by white boxes. (**A**). Proinflammatory cytokine profiles for lungs infected with WU1 cultured in both conditions were compared. (**B**) The mean and standard deviation are shown. Statistical analysis was performed using two-way ANOVA with Fisher’s LSD post-hoc test. *P < 0.05, **P < 0.01, ***P < 0.001, ****P < 0.0001, ns = not significant.

We assessed the histopathological changes (Fig. 7) and cytokine levels (Fig. 8) in each lung lobe following infection with WU1 cultured in SCFM2 vs on agar plates. Overall, we did not observe a major difference between the left and right lobes under each of the specific conditions tested. First, we monitored the effects of the “vehicle controls” and found that administration of 1× PBS led to scores within the normal histological limits, while SCFM2 led to the recruitment of immune cells (Fig. 7A, B, E and F; Fig. S2A, B, E and F). The results of the vehicle control were used as the baseline when assessing the histology of the infected lungs. While WU1 cultured in either SCFM2 or agar plates led to inflammation composed of PMN cells, WU1 cultured in SCFM2 led to a higher degree of inflammation, as seen by the multifocal and coalescing areas of PMN cells in both lung lobes (Fig. 7C, D, G and H; Fig. S2C, D, G and H). Inflammation of lungs infected with WU1 cultured from agar plates was scored as moderate to severe, while lungs infected with WU1 cultured in SCFM2 were scored as severe (Supplemental Table 2).

Next, we measured the cytokine levels in the other lung lobe. Overall, in both lungs, we generally observed higher cytokine levels in lungs infected with WU1 under either growth condition compared with the vehicle controls (Fig. 8). The only exception was a slightly higher level of IL-5 detected in the right lung that was administered the PBS control compared with WU1 cultured on agar plates; however, all these levels were very low. Looking specifically at the right lungs, we observed significantly higher levels of the cytokines IL-6, TNF-⍺, KC/GRO, IL-1β, IL-2, and IL-5 in the mice infected with WU1 cultured in SCFM2 compared with agar plates (Fig. 8B). On the other hand, IL-12p70, IL-10, or IFN-γ had lower or no significant difference between conditions. For the left lungs, significantly higher levels of KC/GRO were detected in the lungs infected with WU1 cultured in SCFM2 compared with agar plates. TNF-⍺, IL-12p70, and IFNγ were significantly lower between conditions, while there were no significant differences in the levels of IL-6, IL-1β, IL-10, IL-2, and IL-5. Overall, we observed a cytokine profile that correlates with the higher levels of inflammation and neutrophil recruitment observed in the histology between the culture conditions. Our findings indicate that culturing WU1 in SCFM2 leads to increased inflammation in both lung lobes, is reflected both in histopathology and cytokine levels.

## DISCUSSION

Laboratory mice have often been used as an *in vivo* model to study *S. aureus* infections, such as bacteremia, sepsis, pneumonia, and many others (5). These models have been essential in advancing our understanding of *S. aureus* virulence and host response but have important limitations. When studying *S. aureus*-induced pneumonia in murine models, the infections are often rapidly cleared (9, 13, 44, 45). Groups have explored the use of different concentrations of inoculum to induce *S. aureus* pneumonia infection. Parker et al. administered 1 × 10^7^ CFUs of *S. aureus* strain USA300 intranasally to C57BL/6J mice and observed a recovery of 0.5 × 10^7^ CFUs from lung homogenates after 24 h (44). Torres et al. observed a similar recovery after administering a lethal dose of 3.78 × 10^8^ CFUs of *S. aureus* strain Newman via the same route to C57BL/6J mice after 18 h (13). Finally, Urso et al. recovered 5 × 10^5^ CFUs of Newman after 24 h following administration of 1 × 10^8^ CFUs intranasally to C57BL/6 mice (45). Additional modifications include using mouse-adapted strains, altered inoculum preparation methods, and depletion of the mouse’s innate immune response (9, 14, 16–19, 46). Here, we hypothesized that culturing *S. aureus* in a media to mimic the lung environment (SCFM2) would increase bacterial loads in the lung.

Our study reveals that increasing *S. aureus* bacterial lung load during a murine respiratory infection relies on the interactions between several factors: the choice of *S. aureus* strain, the choice of mouse strain, and the growth conditions of the bacterial inoculum. Of the six *S. aureus* strains tested, we observed the greatest increase of bacterial lung loads using N315 in C57BL/6 mice and WU1 in Balb/c mice when cultured in SCFM2. WU1’s sustained bacterial loads could be attributed to its host origin (mouse) suggesting it may already be better adapted to evade this specific host immune response. In mice, culturing two commonly studied *S. aureus* strains, JE2 and Newman, in SCFM2 did not increase the bacterial lung loads in the mice while all other strains (Sa_CFBR_43, Sa_CFBR_46, N315, and WU1) showed increased bacterial lung loads. Except for WU1, all *S. aureus* strains tested were isolated from human infections but only strains isolated from respiratory infections (Sa_CFBR_43, Sa_CFBR_46, and N315) demonstrated increased bacterial lung loads when cultured in SCFM2. Interestingly, of the 3 CC8 strains tested, only Sa_CFBR_43 resulted in a significantly higher bacterial lung loads in both mouse strains when cultured in SCFM2 compared with agar plates despite being highly similar genomically to JE2 (99.99%) and Newman (99.85%) based on ANI. When looking at the accessory and unique genes between these strains, our phylogenetic analysis identified several unique genes in Sa_CFBR_43 compared with JE2 and Newman. In future studies, exploring the genetic differences between JE2 and Sa_CFBR_43 might reveal why SCFM2 increases Sa_CFBR_43 bacterial load in mice lungs (20). Sa_CFBR_43 is a CF isolate, and these genetic differences may reflect the adaptation to the lung environment. The impact of strain-specific variation on *S. aureus* virulence during murine pneumonia supports similar results others have observed in murine infection models for bacteremia, abscess, and pneumonia (46–48). In Trübe et al., they observed that a vole-derived CC49 strain was highly virulent in murine pneumonia and bacteremia models. Interestingly, the two CC88 strains they tested had comparable bacterial loads to Newman in their murine pneumonia model. Our results, along with those of others, highlight the importance of testing different *S. aureus* strains when developing murine infection models to account for the differences between strains.

To evaluate the impact of culture conditions on lung bacterial loads across our 6 *S. aureus* strains, we utilized both C57BL/6 and Balb/c mice. These two strains are the most commonly used inbred mouse strains in the field, they are the genetic backbone of many genetically manipulated strains, and they allow the study of different innate immune responses. C57BL/6 mice exhibit a stronger Th1 immune response, while Balb/c exhibit a stronger Th2 immune response (49, 50). Similar studies comparing these two mouse strains in murine bacteremia and skin infections have observed differences in their response to infection (51, 52). Beesetty et al. did compare how C57BL/6 and Balb/c mice respond to pneumonia from USA300 but only focused on survival and not bacterial loads (53). Previous studies of WU1 have primarily focused on its ability to persistently colonize the oropharynx in C57BL/6 mice (20, 42, 43). Our study characterized the ability of WU1 to colonize the lungs of Balb/c mice not only during an acute short-term infection but also during a longer-term infection. Compared with infections in C57BL/6, we found that the bacterial load of WU1 was higher and more consistent in the lungs at 24 h in Balb/c mice. We propose that Balb/c mice may be the preferable choice to study WU1 in the context of bacterial lung loads in SCFM2. Additionally, our findings compared the bacterial load distribution, histology, and cytokine production between the left and right lobes of mice infected with *S. aureus*. The distribution of *S. aureus* to one or the other mouse lung lobe following intranasal administration has not been previously reported according to our literature search. We found that WU1 had similar bacterial loads in both the left and right lung lobes following intranasal infection, as well as similar histopathology scores but different proinflammatory cytokine profiles. When cultured on agar plates, we observed that WU1 bacterial load in the left lung was slightly higher than in the right lung, while the distribution of WU1 cultured in SCFM2 was more similar between lobes. Previous studies had shown that *S. aureus* infections in murine pneumonia trigger inflammation and the recruitment of polymorphonuclear (PMN) cells, and our results were consistent with those findings (9, 30, 54). While both culture conditions resulted in inflammation, the degree of inflammation was higher when WU1 was cultured in SCFM2 compared with agar plates. Interestingly, we observed that administration of SCFM2 alone led to the recruitment of immune cells. Compared with PBS, the other vehicle control, the administration of SCFM2 delivers components that could potentially be more inflammatory, such as mucin and DNA. We speculate that the introduction of potential inflammatory media to the mouse could lead to the observed histological changes. The cytokine profile was generally consistent between the lobes under both culture conditions, but there were some variations. Levels of proinflammatory cytokines (IL-1β, IL-6, TNF-⍺, IFN-𝛾, IL-2, and IL-5) were generally lower in the right lungs compared with the left when infected with WU1 cultured on agar plates but higher when infected with WU1 cultured in SCFM2. In our infection model, WU1, the bacterial loads observed were comparable between the left and right lobes in the lungs, whereas the cytokine response differed between the two, suggesting a divergent host response despite similar bacterial burdens. This suggests that harvesting individual lung lobes may be useful for measuring bacterial load to decrease the number of mice used, but the whole lung may be more appropriate for measuring the host response.

Finally, our results compared how culturing *S. aureus* in SCFM2 vs agar plates affects both respiratory bacterial burden and host response in our *S. aureus* murine pneumonia model. The agar plate preparation has been traditionally used in the laboratory to prepare both *S. aureus* and the bacteria *Pseudomonas aeruginosa* for murine pneumonia infections (55–57). The choice of growing *S. aureus* in SCFM2 in wells was adapted from previous studies that used four-well microchamber slides to study *S. aureus* transcriptomics, physiology, and spatial biogeography (33, 58). A six-well plate format was chosen to maximize the surface area and increase the overall number of *S. aureus*. The comparison between the agar plate, our traditional bacterial method, and the SCFM2 preparation was adapted from a study from our group that compared the transcriptomics of *P. aeruginosa* during murine pneumonia infections (59). However, such a comparison did not exist for *S. aureus*. For all *S. aureus* strains tested here, while SCFM2 can improve bacterial lung load, the overall CFUs recovered from lungs were still lower than the number of originally administered bacteria. This suggests that while *S. aureus* is typically cleared by the host, culturing in SCFM2 may improve the bacteria’s ability to persist beyond 24 h in the lungs.

Overall, we have characterized the potential benefits culturing *S. aureus* in SCFM2 has on bacterial loads during murine respiratory infections as well as the host response compared with LB media. Our study administered *S. aureus* to the mice intranasally but the effect of commonly used methods of administration, such as intratracheal, on the infection will be explored in future studies. Additionally, we report that WU1 infections in the lungs were well maintained up to 4 days post-infection, and future studies will identify whether longer term infection can be achieved. Humanized mice have been shown to be more susceptible to *S. aureus* infections and allowed the study of human response to infection (29, 60). The infection outcome of humanized mice, such as CF humanized mice, when infected with *S. aureus* cultured in SCFM2 might further improve the clinical relevance of this infection model. Finally, while we observed that SCFM2 can increase bacterial loads in the lungs, the mechanisms responsible are not understood. *In vivo* and *in vitro* proteomics and transcriptomics might provide insight into the underlying benefits of SCFM2. Overall, our findings highlight the interplay between bacterial strain, mouse strain, and culture conditions on *S. aureus* ability to colonize during a murine pneumonia model and lay the groundwork for future studies.

## MATERIALS AND METHODS

### Bacterial strain and growth medium selection

Six *S*. *aureus* strains were utilized in this study and listed in Table 1. Lysogeny broth Miller (LB) (BD) was prepared, following the manufacturer’s instructions. LB is a commonly used rich laboratory media composed of tryptone, yeast extract, and sodium chloride. Pre-made SCFM2 was purchased from Synthbiome (31, 32). SCFM2 contains components meant to mimic sputum from patients with CF; these include mucin, DNA, and amino acids.

### Genomic comparative analysis

The sequence assemblies for JE2, Newman, N315, Sa_CFBR_43, and Sa_CFBR_46 were retrieved from NCBI. The whole genome sequence of WU1 was kindly provided by Dr. Dominique Missiakas (University of Chicago). Average nucleotide identities (ANIs) between all assemblies were determined through pyani (61). To identify the core and accessory genome for the strains, a pangenome analysis was performed on the strains. Each strain was annotated with Bakta (62), and the pangenome was estimated with PIRATE v1.0.5 (63). PIRATE was run with default parameters with additional flag -k “--diamond” and -a to use DIAMOND to compare amino acids and obtain core genome alignments, respectively (64). The phylogeny was visualized with Phandango (65) and the R package ggtree (66). Unless stated otherwise, programs were run under default settings. The PIRATE pangenome analysis can be found in Data S1-PIRATE pangenome analysis.

### Bacterial growth curves in LB and SCFM2 in six-well plates

*S. aureus* strains were streaked out on *Staphylococcus* Isolation Agar (SIA) (lysogeny broth [LB] [BD] + 7.5% NaCl). A single colony was transferred to 2 mL of LB and grown overnight at 37℃ shaking at 220 rpm. Overnight cultures of each strain were transferred to a 1.5 mL Eppendorf tube, washed three times with sterile 1× phosphate- buffered saline (PBS), and optical density was measured at 600 nm (OD_600_). The cultures were adjusted to an OD_600_ of 0.01 in 6 mL of LB or pre-warmed SCFM2 and were and grown statically in a six-well plate (Cellstar) at 37℃. Aliquots of each culture were removed at 0, 2, 4, 6, 8, and 24 h, serially diluted, and plated on Lysogeny agar (LA) plates. The plates were incubated at 37℃, and the number of colony-forming units (CFUs) was determined the following day.

### *S. aureus* growth on agar media or SCFM2 workflow

To prepare the *S. aureus* inoculum under our agar plate procedure, strains were streaked on SIA and grown overnight at 37℃ with shaking at 220 rpm. On the following day, a swab of each *S. aureus* was transferred into a sterile Eppendorf tube containing sterile 1× PBS. The strains were washed three times, and OD_600_ was measured. Cultures were adjusted to an OD_600_ of 6.5 in 1 mL of 1× PBS, corresponding to ~1 × 10^8^ CFU per 25-µL dose. An aliquot was removed, serially diluted, and plated on SIA plates to determine CFUs per dose.

For *S. aureus* inoculum preparation in SCFM2, strains were streaked on SIA and grown overnight at 37℃ with shaking at 220 rpm. A single colony was transferred to a culture tube with 2 mL of LB and grown at 37℃ overnight with shaking at 220 rpm. The cultures were then pelleted, washed three times with 1× PBS, and the OD_600_ was measured. The strains were then adjusted to an OD_600_ of 0.01 in 6 mL of pre-warmed SCFM2 in a six-well plate and were grown statically at 37℃ for 24 h. After incubation, the whole well was transferred to a 15-mL Falcon tube, pelleted, and concentrated 15× in fresh SCFM2. An aliquot was removed, serially diluted, and plated on SIA plates to determine CFUs per dose. The bacterial suspension was then immediately utilized for the next steps.

### Mice

All animal experiments were performed according to the guidelines of the Emory University Institutional Animal Care and Use Committee (IACUC) under the approved protocol PROTO201700441. Then, 8–10-week-old female C57BL/6 and Balb/c mice were purchased from Jackson Laboratories (Bar Harbor, ME) and were acclimated for at least a week at Emory University before experiments. All mice were confirmed to be specific pathogen-free at the time of delivery from Jackson Laboratories. Prior to infection, all mice were anesthetized with an intraperitoneal injection of a 0.2-mL mixture of ketamine (6.7 mg/mL) and xylazine (1.3 mg/mL). All mice were euthanized by CO_2_ asphyxiation.

### Lung infection

Anesthetized C57BL/6 and Balb/c mice were intranasally administered 25 µL (12.5 µL per nostril) of each strain cultured either on agar plates or in SCFM2 preparation corresponding to ~1 × 10^8^ CFUs. Mice were humanely euthanized at specified time points, and whole lungs and nasal wash were aseptically collected. For the nasal wash, 1 mL of sterile 1× PBS was flushed through the nasal passage using an 18-G catheter placed at the nasopharyngeal opening. The lungs were weighed and homogenized in 1 mL of sterile 1× PBS inBullet Blender Storm 5 (Next Advance). Both the nasal wash and lungs were serially diluted and plated on SIA. Plates were then incubated at 37℃ overnight, and CFUs were determined.

### Oropharyngeal colonization

Anesthetized Balb/c mice were intranasally administered 25 µL (12.5 µL per nostril) of WU1 prepared either through the agar plate or SCFM2 workflow. To track WU1 colonization in the throat over time, mice were anesthetized with 3% isoflurane through an XGI-8 Gas Anesthesia System (Caliper Life Sciences), and oropharyngeal swabs were collected from each mouse using calcium alginate swabs (Puritan) on days 1, 2, 3, 7, 10, and 14. The swab was cut from the stick with sterile scissors, transferred to a 1.5-mL Eppendorf tube with 500 µL PBS, resuspended, serially diluted, and spread on SIA plates. The plates were then incubated at 37℃ overnight, and CFUs were determined.

### Lung preparation for histology and proinflammatory cytokine analysis

To prepare the left or right lung lobes for histology and cytokine analysis, anesthetized Balb/c mice were intranasally administered 25 µL (12.5 µL per nostril) of *S. aureus* strain WU1 prepared either through the agar plate or SCFM2 workflow. In addition, 1× PBS or SCFM2 were also administered as a “vehicle control.” After 24 h, mice were euthanized, and the chest was opened. A sterile clamp was used to separate the lung lobes; one lobe was inflated with 4% neutral-buffered formalin and removed for histology. The other lobe was processed for cytokine analysis. To determine the bacterial loads in each lung, a parallel group of mice (*n* = 2) was used. Each lobe was separated, weighed, and homogenized in 1 mL of sterile 1× PBS. The lung lobes were plated on SIA, incubated overnight at 37°C, and CFUs were counted.

### Histopathology

Lung specimens from mice were inflated with, and fixed in, 4% neutral-buffered formalin (NBF), processed, and blocked in paraffin for histological analysis. All samples were sectioned at 5 µm and stained with hematoxylin-eosin (H&E) for routine histopathology. Samples were evaluated by a board-certified veterinary pathologist in a blinded manner. Scoring was determined by the proportion (%) of the affected lung tissue: - = no evidence of inflammation; +/− (10%–20%) minimal to mild; + (20%–50%) mild-moderate; ++ (50%–75%) moderate-severe; and +++ (<75%) severe (consolidated areas present). Sections were examined under light microscopy using an Olympus BX51 microscope, and photographs were taken using an Olympus DP73 camera. Images were taken at either a 4×, 5×, or 6× magnification.

### Cytokine analysis

Lung lobes were weighed, homogenized in 1 mL of sterile 1× PBS supplemented with ProBlock Protease Inhibitor Cocktail (Goldbio), the supernatant was collected through centrifugation, and stored at −80℃. Cytokine levels were measured through the MesoScale Discovery (MSD) PlatformV-PLEX proinflammatory Panel 1 Mouse Kit. The cytokines measured through this kit were IFN-𝛾, IL-1β, IL-2, IL-4, IL-5, IL-6, IL-10, IL-12p70, KC/GRO, and TNF-⍺. The cytokine levels were quantified and analyzed at the Emory Multiplex Immunoassay Core with MESO QuickPlex SQ 120, following the manufacturer’s instructions. Results were normalized to lung weight. Results for IL-4 were not included as the results from all groups were below the limit of detection.

### Statistical analysis

Statistical analyses were performed for all experiments using GraphPad Prism 9.

## Data Availability

The genomic sequences of JE2 (BioProject PRJNA381486), Newman (BioProject PRJDA18801), N315 (BioProject PRJNA264), Sa_CFBR_43 (BioProject PRJNA480016), and Sa_CFBR_46 (BioProject PRJNA480016) are available on NCBI. WU1’s (https://doi.org/10.6082/jzw48-vnn40) genomic sequence is available on UChicago Knowledge.

## SUPPLEMENTAL MATERIAL

Data S1 iai.00283-26-s0001.xlsx Pirate pangenome analysis.

Supplemental material iai.00283-26-s0002.pdf Tables S1 and S2; Fig. S1 and S2.

ASM does not own the copyrights to Supplemental Material that may be linked to, or accessed through, an article. The authors have granted ASM a non-exclusive, world-wide license to publish the Supplemental Material files. Please contact the corresponding author directly for reuse.
