# Supplementary material for: Growing Staphylococcus aureus in synthetic cystic fibrosis medium promotes colonization in a murine pneumonia model
Source: Infect Immun. 2026 Aug 6;94(9):e00283-26. doi: 10.1128/iai.00283-26 (PMC13552117; doi:10.1128/iai.00283-26)
Supplement: Supplementary file 1 [file iai.00283-26-s0002.pdf]

SUPPLEMENTAL MATERIALS

Growing *Staphylococcus aureus* in Synthetic Cystic Fibrosis Medium Promotes Colonization in a Murine Pneumonia Model

Justin M. Luu[a,b], Ian N. Moore[c], Dina A. Moustafa[b,d], and Joanna B. Goldberg[b,d*]

[a]Microbiology and Molecular Genetics Program, Graduate Division of Biological and Biomedical Sciences, Laney Graduate School, Emory University, Atlanta, Georgia, USA

[b]Department of Pediatrics, Division of Pulmonary, Asthma, Cystic Fibrosis, and Sleep, Emory University School of Medicine, Atlanta, Georgia, USA

[c]Department of Pathology and Laboratory Medicine, Emory University School of Medicine, Atlanta, Georgia, USA

[d]Emory+Children's Center for Cystic Fibrosis and Airway Disease Research, Emory University School of Medicine, Atlanta, Georgia, USA

Table S1. Average nucleotide identity values.

Strain	JE2	N315	Newman	Sa_CFBR_43	Sa_CFBR_46	WU1
JE2	1	0.98950869	0.999075822	0.99987798	0.973625079	0.990052994
N315	0.989491561	1	0.9894661	0.989440433	0.973406043	0.989826162
Newman	0.999024674	0.989454179	1	0.99848061	0.973404448	0.99000707
Sa_CFBR_43	0.99989204	0.98936415	0.998515885	1	0.973239822	0.990193949
Sa_CFBR_46	0.973552667	0.973405941	0.973365271	0.973253801	1	0.973250535
WU1	0.99007895	0.989852571	0.990038341	0.990103116	0.973251346	1

Average nucleotide identity (ANI) was determined between the genomic sequences of each strain using pyani. The table depicts the numerical value outputs from the analysis.

Table S2. Histology Scores.

Left Lung Lobe Condition	Score	Right Lung Lobe Condition	Score
PBS	-	PBS	-
SCFM2	+/-	SCFM2	+/-
WU1 (Agar)	++	WU1 (Agar)	++
WU1 (SCFM2)	+++	WU1 (SCFM2)	+++

Each lung was blindly scored by a veterinarian pathologist. Scoring was determined by the proportion (%) of the affected lung tissue: - = no evidence of inflammation; +/- (10-20%) minimal to mild; + (20-50%) mild-moderate; ++ (50-75%) moderate-severe; and +++ (<75%) severe (consolidated areas present).

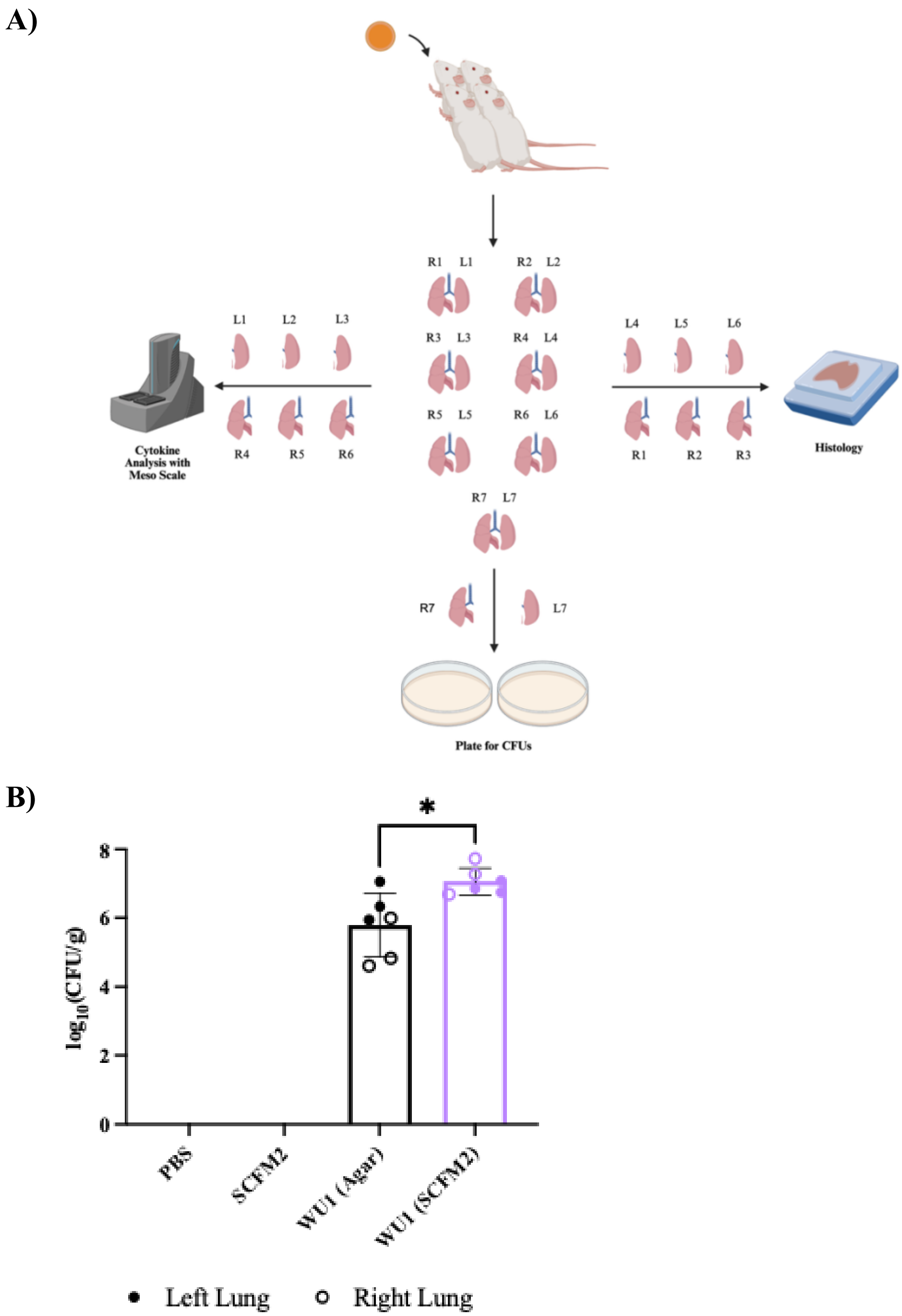
A)
R1 L1
R2 L2
R3 L3
R4 L4
R5 L5
R6 L6
R7 L7
L1 L2 L3
R4 R5 R6
L4 L5 L6
R1 R2 R3
Cytokine Analysis with Meso Scale
Histology
R7
L7
Plate for CFUs
B)
*
log10(CFU/g)
8
6
4
2
0
PBS
SCFM2
WU1 (Agar)
WU1 (SCFM2)
Left Lung
Right Lung

Supplemental Figure 1. Workflow for lung lobe separation for cytokine analysis and histology.

A) Seven 8–10-week-old Balb/c mice were used for each culture condition (Agar vs. SCFM2). Mice were euthanized 24 hours post infection and for each mouse, a clamp was inserted to separate the left and right lobes. For 3 mice, one lobe was inflated with 4% neutral-buffered formalin (NBF), separated, and stored in 4% NBF for histology. The other lung lobe was homogenized in 1X PBS + 1X protease inhibitor, the homogenate supernatant was isolated, and cytokines were measured with the V-plex proinflammatory cytokine panel 1 (MSD). In 3 other mice, the alternate lung lobe was process as described above leading to 3 left and 3 right lung lobes from different mice for histology and cytokine analysis. **B)** The left (filled symbol) and right (open symbol) lobe from the 7th mouse was used to determine CFUs as described above. Statistical analysis was performed using two-way ANOVA with Fisher's LSD post-hoc test. $*p < 0.05$.

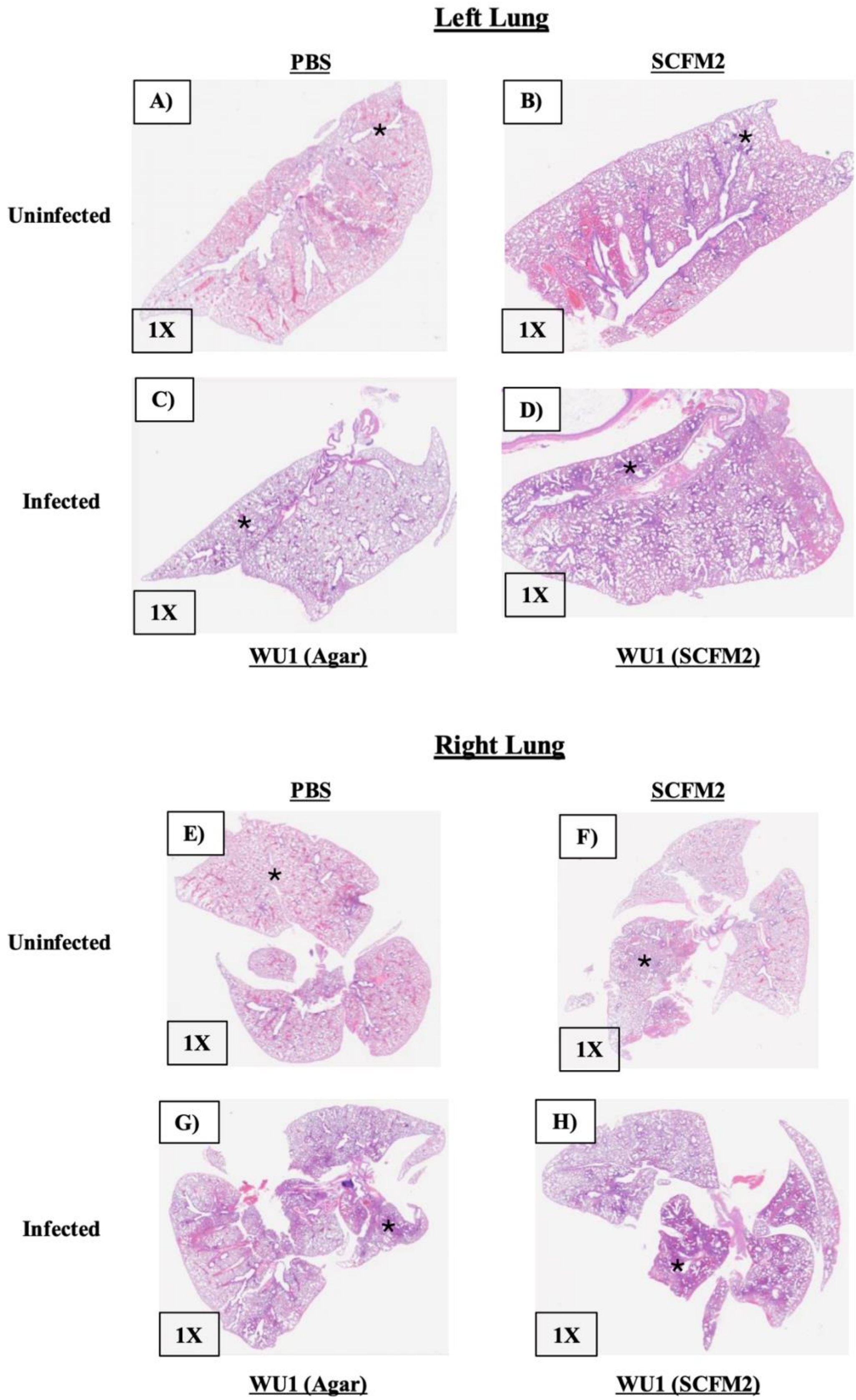
Left Lung
PBS
SCFM2
A)
B)
Uninfected
1X
1X
C)
D)
Infected
1X
1X
WU1 (Agar)
WU1 (SCFM2)
Right Lung
PBS
SCFM2
E)
F)
Uninfected
1X
1X
G)
H)
Infected
1X
1X
WU1 (Agar)
WU1 (SCFM2)

Supplemental Figure 2. 1X magnification of lungs infected with WU1 cultured on agar plates or in SCFM2.

S. aureus strain WU1, cultured either on agar plates or in SCFM2 as described in the workflow in Figure 3, was administered to 8–10-week-old female Balb/c mice. Mice were euthanized 24 hours post infection. For each culture workflow (Supplemental Figure S1) the left lung **(A-D)** or right lung **(E-H)** lobes from each mouse (n=6) were inflated and fixed using 4% NBF. The lungs were then processed, strained with H&E, and blindly assessed by a veterinary pathologist. A representative image for each lung lobe is shown at 1X magnification. * indicates the regions that were magnified in Figure 7.
